# Genome-wide analysis of intraspecific transposon diversity in yeast

**DOI:** 10.1186/1471-2164-14-399

**Published:** 2013-06-14

**Authors:** Claudine Bleykasten-Grosshans, Anne Friedrich, Joseph Schacherer

**Affiliations:** 1 CNRS, Department of Genetics, Genomics and Microbiology, University of Strasbourg, UMR 7156, 28, rue Goethe, Strasbourg, 67083, France

**Keywords:** Transposons, Retrotransposons, *Ty* elements, *Ty1*, Intra-specific diversity, Genome evolution, *Saccharomyces cerevisiae*

## Abstract

**Background:**

In the model organism *Saccharomyces cerevisiae*, the transposable elements (TEs) consist of LTR (Long Terminal Repeat) retrotransposons called *Ty* elements belonging to five families, *Ty1* to *Ty5*. They take the form of either full-length coding elements or non-coding solo-LTRs corresponding to remnants of former transposition events. Although the biological features of *Ty* elements have been studied in detail in *S. cerevisiae* and the *Ty* content of the reference strain (S288c) was accurately annotated, the *Ty*-related intra-specific diversity has not been closely investigated so far.

**Results:**

In this study, we investigated the *Ty* contents of 41 available genomes of isolated *S. cerevisiae* strains of diverse geographical and ecological origins. The strains were compared in terms of the number of *Ty* copies, the content of the potential transpositionally active elements and the genomic insertion maps. The strain repertoires were also investigated in the closely related *Ty1* and *Ty2* families and subfamilies.

**Conclusions:**

This is the first genome-wide analysis of the diversity associated to the *Ty* elements, carried out for a large set of *S. cerevisiae* strains. The results of the present analyses suggest that the current *Ty*-related polymorphism has resulted from multiple causes such as differences between strains, between *Ty* families and over time, in the recent transpositional activity of *Ty* elements. Some new *Ty1* variants were also identified, and we have established that *Ty1* variants have different patterns of distribution among strains, which further contributes to the strain diversity.

## Background

Transposable elements (TEs) are interspersed repetitive and mobile DNA sequences. They exist in almost all the eukaryotic genomes characterized so far, where they often constitute the largest component. TEs belong to two classes, depending on whether the RNA-mediated ‘copy and paste’ (class I) or DNA-mediated ‘cut and paste’ (class II) mode of transposition is involved. As the result of their ability to proliferate and move to different positions, they give rise to inter- and intra-species genomic differences. The mutational activities of TEs, which result in gene disruption and chromosome rearrangements, also contribute to their hosts’ genetic and phenotypic diversity [1-3]. TE insertions can actually result in novel specific patterns of gene expression by either acting as regulatory elements or disrupting these elements. In addition, TEs can serve as targets for epigenetic modifications [4]. TE induced diversity may therefore have much more complex phenotypic effects than those resulting from point mutations. Since selection processes can operate on TE induced variations, TEs are thought to be particularly powerful agents responsible for adaptive changes and strong drivers of genome evolution [5]. TE activation or reactivation is likely to greatly affect genome evolution, which raises questions as to the difference in “evolvability” existing between organisms showing variable TE contents and even between isolates belonging to the same species.

There exist some extremely marked differences between the TE contents of various species, in terms of the number of copies, the TE repertoires and the respective proportions of full-length, mutated and fossil copies present [6,7]. These differences result partly from host traits (such as their regulation and defense strategies), and partly from features of TEs themselves, which are responsible for their expansion, persistence and extinction in genomes. It has been established that the TE contents of genomes vary not only between species, but also in some cases between populations belonging to the same species [8]. The polymorphism of TE is therefore widely used as an indicator to assess the genetic diversity of organisms with moderate to high TE contents: diagnostic insertion profiles are generated using PCR-based methods such as the Transposon Display, IRAP (Inter-Retrotransposon Amplified Polymorphism) and RBIP (Retrotransposon Based Insertion Polymorphism) methods [9] to obtain phylogenetic markers that are commonly used for mapping, genotyping and taxonomic purposes [10-14]. Since the advent of new sequencing technologies, new approaches have been developed for detecting insertion polymorphism, such as those based on targeted sequencing of TE junctions via sequence capture enrichment procedures [15] and *in silico* methods designed for investigating sequencing data and genome assemblies in repetitive elements [16-18].

Apart from multicellular organisms, some eucaryotes have relatively poor TE contents. It has been suggested, for example, that hemiascomycetous yeast may have undergone massive TE losses [19], since the TE fraction does not occur in more than 5% of their genomes, and the TE classes and families show a patchy distribution among ‘reservoir species’ and apparently ‘empty species’. *Candida albicans* harbors many potentially active copies of TE belonging to various families of class I and class II elements, for example, whereas closely related species such as *Candida glabrata* and *Pichia sorbitophila* carry only a few degenerate copies and show no traces at all of TEs, respectively [20]. Whether these TE landscapes are characteristic of the species as a whole, rather than being restricted to the strains that were sequenced, still remains to be established by investigating the TE content of a large number of isolates. The inter- and intra-species TE polymorphism has also been previously used to typify industrial strains [21] and to detect some noteworthy aspects of both the evolution of yeast TE and the strain diversity [22-24].

LTR (Long Terminal Repeat) retrotransposons are the main TEs occurring in hemiascomycetes. The putative origins of the present LTR retrotransposon repertoire have been deduced from both the structural features and the inter-species distribution of these elements [22,25,26]. Their evolutionary scenario has been mostly drawn up assuming the occurrence of a process of vertical transmission. *Gypsy*-like elements are present in all the species in the Hemiascomycete phylum and therefore seem to be the most ancient acquisition [19]; whereas the phylogeny of the elements belonging to the four lineages of *Copia*-elements (*Ty1, Tca2, Ty4* and *Ty5*) is almost identical to that of their hemiascomycetous hosts [25]. The various *Copia*-element families may therefore have evolved as the result of successive radiation events from a single ancestral family resembling the present *Ty5* elements. Starting with *Ty5*-like elements encoding a single ORF, some novel features such as an in-frame stop codon (in the *Tca2* lineage), a programmed frameshift between the *TYA* and *TYB* genes (in the *Ty1* lineage) and a change of primer binding site (in the *Ty4* lineage) were acquired during the speciation steps. The structural characteristics and the pattern of host distribution of the elements belonging to the *Ty4* lineage suggest that these are the youngest elements [19,25]. The possibility that horizontal transmission events may also have occurred was suggested in the case of two elements belonging to the *Ty1* lineage: *Tsk1* in *Lachancea kluyveri*[25] and *Ty2* in *Saccharomyces cerevisiae*[22,26].

In comparison with its nearest hemiascomycetous relatives, the model species *S. cerevisiae* is thought to constitute an exceptional TE reservoir. Only LTR-retrotransposons are present in this species, but it contains more *Ty* families with full-length copies than other yeast species [20]. These elements called *Ty* elements account for 1.5% of the genome. Most of the known aspects of LTR-retrotransposon biology have been discovered by studying the *Ty* elements present in *S. cerevisiae*[27]. These *Ty* elements belong to five families, *Ty1* to *Ty5*. *Ty3* is a *Gypsy*-like retrotransposon and *Ty1*, *Ty2*, *Ty4* and *Ty5* are *Copia*-like retrotransposons. In the reference genome of the S288c strain, the organization of the *Ty* elements among potentially full-length active copies and solo-LTR resulting from inter-LTR recombination has been thoroughly annotated and described [26,28]. In this strain, the *Ty1* family is the largest and most active one: it contains 32 full-length copies and more than 250 solo-LTRs. Previous studies [29-32] and analyses on the genome sequences of several additional *S. cerevisiae* strains [33-38] have clearly shown the existence of differences in the *Ty* localization, the number of copies and the relative size of *Ty* families, depending on the genetic background involved. However, except for the strain K7, these analyses have been restricted to full-length *Ty* elements and no systematic comparative surveys of the complete *Ty* landscape associated with solo-LTR elements have yet been carried out with a view to further understanding how *Ty* elements have contributed to the genotypic and phenotypic diversity of *S. cerevisiae*.

The complete genome sequences of 41 *S. cerevisiae* isolates available were therefore used in this study to examine and compare the *Ty*-related elements occurring in a whole species. The number of copies and the genomic locations of all the *Ty* elements were determined. The resulting overall picture yielded some interesting clues about the evolutionary history of *Ty*-related polymorphism. We also detected new *Ty1* variants, and observed that the repertoires of subfamilies corresponding to the closely related *Ty1* and *Ty2* elements differ from one strain to another.

## Results and discussion

### Genome-wide detection of *Ty* elements in various *S. cerevisiae* genetic backgrounds

The genomic assemblies available for 41 *S. cerevisiae* strains were sampled: these consisted of the reference strain S288c and a set of 40 additional strains covering a broad range of ecological and geographical origins (Table 1). The large range of geographical origins and habitats was previously found to be associated with considerable genomic variability in the single nucleotide polymorphism (SNP) of the strains [39], which raised questions about the variability of the *Ty* elements present in these strains.

**Table 1:** Strains investigated in this study

**Strain**	**Location**	**Source**	**Reference**
AWRI1631	South Africa	Wine	[40]
AWRI796		Wine	[40]
CBS7960	Brazil	Bioethanol	*
CLIB215	New Zeland	Baker	*
CLIB324	Vietnam	Baker	*
CLIB382	Ireland	Beer	*
EC1118	France	Wine	[35]
FL100		Laboratory	*
FOSTERSB		Beer (ale)	[40]
FOSTERSO		Beer (ale)	[40]
I14	Italy	Vineyard (soil)	*
IL01	US	Nature (soil)	*
JAY291	Brazil	Bioethanol	[36]
LALVINQA23		Wine	[40]
M22	Italy	Vineyard	*
NC02	US	Nature (tree exudate)	*
PW5	Nigeria	Fermention (palm wine)	*
RM11	US	Wine	[41]
S288C	US	Laboratory	**
SIGMA1278		Laboratory	[42]
SK1		Laboratory	[43]
T73	Spain	Wine	*
T7	US	Nature (tree exudate)	*
UC5	Japan	Sake	*
VIN13		Vineyard	[40]
VL3		Wine	[40]
WE372	South Africa	Wine	*
Y10	Philipines	Fermentation (coconut)	*
Y12	Ivory Coast	Fermentation (palm wine)	*
Y9	Indonesia	Fermentation (ragi)	*
YJM269		Fermentation (apple juice)	*
YJM280	US	Clinical	*
YJM320	US	Clinical	*
YJM326	US	Clinical	*
YJM421	US	Clinical	*
YJM428	US	Clinical	*
YJM451	US	Clinical	*
YJM653	US	Clinical	*
YJM789	US	Clinical	[33]
YPS1009	US	Nature (oak exudate)	*
YPS163	US	Nature (oak exudate)	*

* http://www.genetics.wustl.edu/jflab/data4.html.

** http://www.yeastgenome.org/.

### Variability of the LTR contents

**Figure 1:**
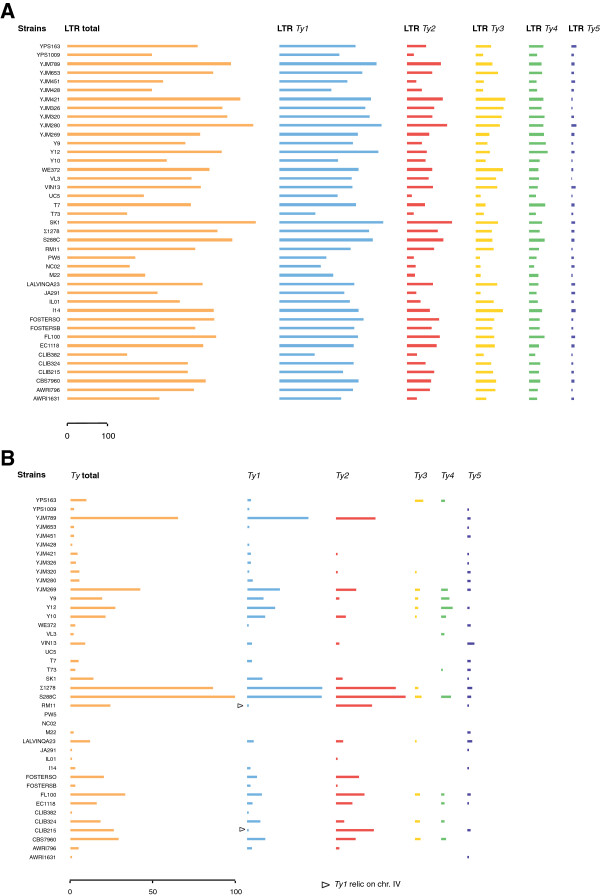
**Differences between strains in the LTR contents.** The size of the bars indicates the total number of LTR copies in each strain, and in each *Ty1* to *Ty5* family. **A**) All the LTRs detected **B**) LTRs belonging to *Ty* coding-elements. The arrowheads indicate the *Ty1* relic copies.

Since LTRs are the most abundant *Ty* sequences in the reference S288c genome, LTR sequences from *Ty1* to *Ty5* elements were used as query sequences to screen the 41 genomic sequences. The query sequences were chosen from representative transposition competent elements belonging to each *Ty* family (Additional file 1). The number of LTR sequences detected in each strain and their distribution among *Ty* families were determined (Figure 1A and Additional file 2: Table S1). The total number of LTRs detected was found to range from 147 (in the strain T73) to 463 (in the strain SK1), giving a mean number of 315 elements. No clear-cut correlations were detected between the LTR contents and the ecological and geographical origins of the strains, but only a slight bias in the case of the laboratory and clinical strains, which showed the highest LTR contents. The *Ty1* LTRs are the most abundant, accounting for 59% of the elements detected both on average and in each individual strain. These results show that LTR sequences belonging to all five *Ty* families are present and have accumulated in all the strains investigated here. If we take the present number of LTR copies to be an indicator of former transpositional activity, the *Ty1* elements can be said to be the most transpositionally active elements in the *S. cerevisiae* species as a whole. The three-fold difference observed between the maximum and minimum number of LTR copies suggests that the transpositional activity responsible for the process of LTR accumulation observed differs from one strain to another. However, the differences between the strains investigated here were far from being comparable to those observed between the *tirant* LTR retrotransposon and the *helena* non-LTR retrotransposon in *Drosophila simulans* populations [44,45] and between the *mPing* MITE transposons in various rice strains [46]. Alternatively, some strains may have undergone intense transpositional activities, but if their LTR elements are highly fragmented as the result of successive nested insertions, they may have escaped both resolution during the steps generating the genomic assemblies and detection by our searches. However the latter point cannot be addressed without finishing the genomic sequences manually.

### Variability of the *Ty* coding-element contents

Previous authors have described the differences between strains in terms of the *Ty* coding-elements they contain [33-36]. In the reference genome S288c, only 15% of the *Ty* sequences were found to be full-length *Ty* elements encoding the proteins TyA (Gag) and TyB (Pol). The remaining sequences correspond to solo-LTRs, which result from inter-LTR recombination events. We therefore investigated the contents of the various genomes in terms of their potentially full-length active *Ty* element contents. For this purpose, the adjacent LTR sequences were extracted and screened to detect the presence of either TyA or TyB coding sequences in the appropriate orientation. The resulting data show that only 5% of the LTRs detected belong to *Ty* coding-elements (Figure 1B, Additional file 2: Table S1). Contrary to the overall LTR contents, the abundance of the LTRs belonging to *Ty* coding-elements is highly variable among the strains (Additional file 3: Figure S1) and only weakly correlated with the total number of LTR copies (Additional file 4: Figure S2, R = 0.415). This variability was observed at several levels. First, the two strains S288c and SIGMA1278 contain a remarkably large number of LTRs corresponding to *Ty* coding-elements amounting to 99 and 86 copies, respectively, which account for 30% of all the LTRs belonging to coding-elements. Secondly, three strains (NC02, PW5, UC5) have no LTRs belonging to *Ty* coding-elements. Segments of coding-elements were detected, however, in the genome assemblies of these strains when *TYA* and *TYB* sequences were used as query sequences, which suggests that these strains may carry very few intact *Ty* elements (Additional file 2: Table S1). Lastly, only 21 of the remaining strains have more than five LTR copies corresponding to *Ty* coding-elements. In the strains showing very few *Ty* coding-elements, either the transpositional activity responsible for the previous process of LTR accumulation has decreased or the present transpositional activity does not suffice to counterbalance the loss of full-length elements resulting from inter-LTR recombination events. Importantly, we have checked that there is no correlation between the contents in *Ty* coding-elements and the quality criteria of the surveyed genome assemblies (Additional file 5: Table S2). All the genome assemblies studied here result from sequencing methods generating reads, which size exceeds the size of a single *Ty* LTR (Additional file 5: Table S2). Nevertheless, for three strains (CLIB382, NC02 and YJM428), one should not exclude that very few coding-*Ty* have been detected because of the particularly low quality of their assemblies (more than 10,000 scaffolds).

On average, the proportion of LTRs observed in the *Ty* coding-elements belonging to families *Ty1* to *Ty5* was the same as in the S288c reference strain (43%, 39%, 5%, 6% and 2%, respectively). The rates of occurrence of LTRs in *Ty5* coding-elements are slightly higher (7% on average). In many of the individual strains, however, the above proportions between *Ty* families were no longer observed. This is partly due to the fact that in several strains, some *Ty* families lack LTRs belonging to coding-elements. As previously described in the strains YJM789 and EC1118 [33,35], for example, 24 additional strains may lack either *Ty3* or *Ty4* coding-elements, or both. Another example is given by the *Ty1*/*Ty2* ratio, which is commonly used to compare yeast strains [35,47,48]. Among the strains investigated here, this ratio was found to be variable, either due to no *Ty2* coding-elements being detected (in strains CLIB382, I14 and Y12) or to the prevalence of *Ty2* full-length elements over *Ty1* elements (FOSTERSO, RM11, FL100, CLIB215, IL01 and CLIB382 strains). It is worth noting that the sole *Ty1* coding-element detected in RM11 and CLIB215 is in fact an inactive relic (see below), which indicates that *Ty1* may be extinct in these two strains. However, the LTR contents attest that *Ty1* was recently an active *Ty* family.

Importantly, the highly variable *Ty* coding-element contents result in differences in the future *Ty* expansions among the various isolates. The existence of ‘*Ty* permissive’ strains, in which full-length potentially functional *Ty* elements have subsisted, and ‘non *Ty* permissive’ strains, which are poorly endowed or even devoid of functional *Ty* elements raises several questions. (i) Are the differences in the *Ty* coding-elements due to a recent decrease in transpositional activity or to an enhanced host response, leading to the loss of *Ty* coding-elements? Interestingly, it was reported in a previous study that the ‘*Ty* permissive’ strain FL100 showed greater transpositional activity than the ‘*Ty* permissive’ strain S288c, which suggests that the mechanism involved in *Ty* maintenance may depend on the genetic background [49]. (ii) May the differences in *Ty* content result from differences between the genetic backgrounds rather than depending on the strains’ preponderant state of propagation (haploid or diploid) or their ecological niches? If so, what are the genetic determinants responsible for the maintenance/deletion rates of functional *Ty* elements? (iii) *Ty* elements are known to be stress sensitive [27], and it has been hypothesized that populations showing enhanced TE activity are more likely to survive during environmental fluctuations because they produce a larger number of genomic variants for natural selection processes to work on [50]. It would therefore be interesting to compare the adaptive potential of these strains: how do the various *Ty* contents affect the adaptation processes and how are they themselves influenced during these processes?

### Genome-wide distribution of the *Ty* elements

Genomes differ not only in their TE content but also in the location of their TE insertions, resulting in different maps of occupied and empty loci. In order to assess the intra-specific polymorphism of *Ty* insertions, we extracted the neighboring sequences with respect to the *Ty* elements detected (either LTRs, corresponding to all the *Ty* insertions, or *Ty* coding-elements) and mapped them against the S288c reference genome (Additional file 6: Figure S3). It is worth noting that the distributions of the *Ty*-related insertions detected are consistent with their respective target preferences. *Ty1* to *Ty4* show a preference for insertion points located near genes transcribed by RNA polymerase III [28,51]. Genes of this kind were found to be present in the flanking sequence of 62% of the elements detected (Additional file 2: Table S1).

Different ways of presenting the resulting data illustrate the various aspects of the polymorphism of *Ty* insertions. At each *Ty* insertion locus, we sought to determine whether its occupancy was specific to a given strain or whether it also occurred in other strains. The insertion maps give the number of strains showing a *Ty* insertion belonging to the same family at the same locus (Additional file 6: Figure S3). This number ranged between one and 41 strains, depending on the locus. The maps show nearly homogeneous patterns of inter-chromosomic distribution between singly and shared occupied loci, regardless of the *Ty* family involved. Two noticeable exceptions were observed, however: chromosome XI does not carry any highly shared loci in the case of the families *Ty2*, *Ty3* and *Ty4*, and chromosome XV does not carry any highly shared loci in the case of the *Ty4* family. Highly shared loci may correspond to fixed TE insertions occurring either early during the host’s evolution and/or as the result of positive selection processes.

Focusing on *Ty* coding-elements, we observed that contrary to what occurs with the LTRs, the patterns of locus occupancy are mostly either specific to a given strain or shared by just a few strains (only 18 out of the 130 occupied loci are common to more than five strains). The insertion maps are therefore potentially more polymorphic in the case of *Ty* coding-elements than in that of LTRs (see below). Few of the loci occupied by a *Ty* coding-element insertion in a given strain also carry an LTR insertion in the other strains. This finding indicates that recent strain-specific transposition events were the main cause of the insertional polymorphism observed. It also suggests that the loss of *Ty* coding-elements mediated by inter-LTR recombination events occurred early after the transpositional insertion process. Interestingly, the two *Ty* coding-element insertions that are common to the largest number of strains are non-functional relics of *Ty1* coding-elements located on chromosome IV (one of which was mentioned above, and will be referred to again below). Both copies may encode the TyA protein but lack the *TYB* gene and the terminal LTR, which may have enabled the excision of their coding region to occur.

**Figure 2:**
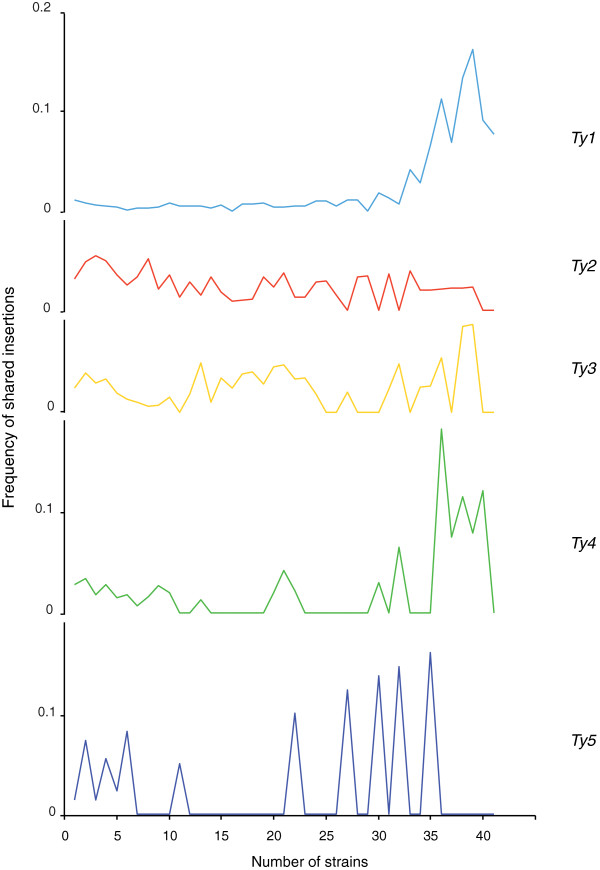
**Patterns of insertional polymorphism of the *Ty1 *to *Ty5 *families.** The number of loci occupied by an LTR insertion was plotted against the number of strains having insertions at the same locus, normalized by the total number of insertions corresponding to each *Ty* family.

In addition, the loci containing *Ty1* insertions are occupied by a larger number of strains (20 strains on average) than the loci where the insertions belong to other *Ty* families (Additional file 6: Figure S3). The occupancy of the loci containing *Ty2* is that which occurs the least commonly among the strains (involving only 7 strains on average). Half of the loci containing *Ty1* were observed, for example, in more than 20 other strains, whereas only 11 to 22% of the loci occupied by the families *Ty2* to *Ty5* are common to more than 20 strains. These differences between *Ty* families were presented by plotting the number of insertions against the number of strains showing these same insertions at the same locus, normalized by the total number of insertions observed in the whole *Ty* family (Figure 2). This yielded characteristic patterns reflecting the level of polymorphism of each *Ty* family. The *Ty1* pattern is characterized by a low level of polymorphism between individuals because almost all the same loci are occupied in many strains. By contrast, the *Ty2* pattern observed shows that the insertions are equally distributed between single loci and loci with medium and high rates of common occupancy, and the *Ty3* insertions preferentially show medium rates of common occupancy. In the case of *Ty4*, most of the insertions are highly shared, but there are also considerable numbers of single and medium rates of occurrence of common insertion. *Ty5* insertions occur at either single or common loci, but the small number of insertions observed (26) makes it difficult to detect a significant pattern of distribution.

**Figure 3:**
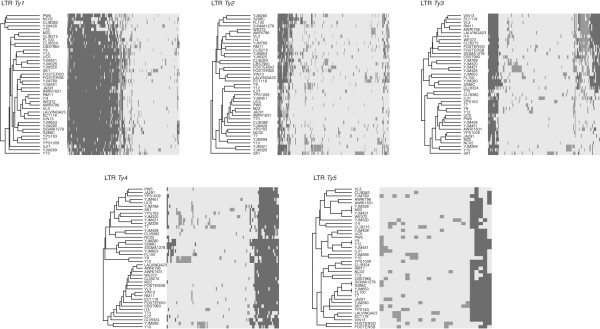
**Differences between strains in the locations of LTR insertions.** LTR insertion profiles of *Ty1* to *Ty5* families: in each strain, each grey rectangle indicates the presence of a *Ty* insertion at the corresponding locus. Dark grey rectangles indicate insertions in common with the S288c reference strain. Hierarchical clustering analysis was applied to both the strains and the loci. The resulting trees are presented in the case of the strains.

Lastly, in order to show up the likenesses and differences between strains, we compared their profiles of locus occupancy (Figure 3). These profiles were drawn up on the basis of presence/absence matrices (Additional file 7) in the case of both the LTRs (Figure 3) and the coding elements present in each *Ty* family (Additional file 8: Figure S4). TE distributions are assumed to recapitulate both the history of the TE family and the evolution of the host [50]. TE insertions (and particularly retrotransposon insertions) are therefore widely used as genetic markers for studying evolutionary and population relationships. This emerged particularly clearly here upon looking at the common insertions detected between the closely related lab strains S288c, FL100 and SIGMA1278 (Figure 3 and Additional file 8: Figure S4) [52]. In this context, the fact that the insertion profiles of a given TE family are very similar in all the strains may result from the presence of fixed insertions and reflects the fact that few new insertions have occurred since the divergence of the strains. However, the *Ty1* LTR profiles cannot be interpreted on these lines. It is known from the S288c reference strain that at a given insertion locus, several *Ty1* insertions are often adjacent and even nested, whereas the insertions from the other *Ty* families are considerably less numerous and more widely dispersed. The low level of polymorphism observed among *Ty1* occupied loci (Figure 2) and the large number of loci common to many strains may therefore result here from the saturation of the *Ty1* insertion sites rather than from the presence of fixed insertions. It can be seen from the *Ty2* LTR insertion profiles that these sites are the most variable among strains, reflecting a later and probably still ongoing period of activity. These findings are consistent with (i) the hypothesis that *S. cerevisiae Ty2* elements have been recently acquired [22,26] and with (ii) the fact that, at least in some strains, *Ty2*-related sequences are transposed in the form of *Ty1/2* hybrids (see below). In the case of these hybrid elements, the transcription rates recorded in [53] and the transposition rates in the S288c background recorded in [54] are particularly high. The profiles of the *Ty4*-related *loci* are those showing the largest numbers of insertions common to many strains (Figure 3). However, some strains carry additional insertions (Y9, Y12, Y10, YJM269 and SK1), which suggests that *Ty4* activity occurred later on. The *Ty3* and *Ty5* profiles are the most highly structured ones: the insertion profiles of loci with mean rates of shared occupancy reflect the presence of clearly visible strain clusters. These clusters may have resulted from a period of activity of *Ty3* and *Ty5* elements that took place after the *Ty4* expansion and before the *Ty2* expansion. Both the *Ty3* and *Ty4* profiles suggest that they resulted from several waves of amplification/activation subsequent to periods of inactivity. It was previously suggested that this behavior might explain the distribution of the LTR-retrotransposon families observed in the rice genome [55].

It has been stated that the majority of the *Ty* insertions are fixed and that the catalog of *Ty* insertion sites discovered using the S288c reference genome describes the core state of most *Ty* element locations across strains in *S. cerevisiae*[26]. The comparisons made here between insertion profiles clearly show which insertions are specific to each of the strains investigated. For example, the strain SK1 was found to have a remarkably large number of *Ty1*, *Ty2* and *Ty3* insertions, which is consistent with previous data on its SNP polymorphism [39]. IL01 carries a specific set of *Ty3* insertions and Y9 and Y12 have particular *Ty4* insertion profiles. It would be interesting to know whether these *Ty* amplifications may have an impact on the phenotypic diversity of these isolates.

### Variability of the *Ty1* and *Ty2* coding-elements

**Figure 4:**
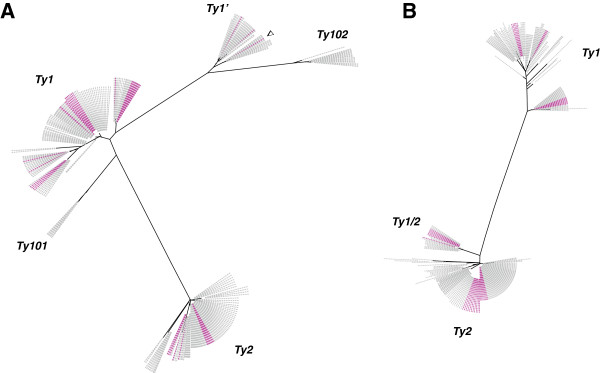
***Ty1 *and *Ty2 *coding-element subfamilies.** Phylogenic trees were drawn up, based on 300 aligned nucleotide positions. Individual sequence names have been omitted. Branches are drawn to scale. Pink-colored leaves correspond to sequences detected in the strain S288c. Grey leaves correspond to sequences detected in all the remaining strains. The corresponding *Ty* family or subfamily is indicated for the clusters. The arrowhead indicates the *Ty1’* relic copy. **A**) TYA300 neighbor joining tree based on the 300 nucleotides, in line with the 5′ LTR and the distribution of elements in the strain S288c. **B**) TYB300 neighbor joining tree based on the 300 nucleotides preceding the 3′ LTR and the distribution of elements in the strain S288c.

TE copies of the same element are not identical and the diversity of the TEs themselves may contribute to the strain diversity. *Ty1* and *Ty2* related sequences are the most abundant sequences detected in the strains investigated here. Based on phylogenetic data, these two families have been found to be closely related [25]. However, their coexistence in *S. cerevisiae* does not result from a *Ty* speciation process occurring in the same host, but *Ty2* may have been acquired via a process of horizontal transfer from the *S*. *mikatae* species [22,26]. The most suitable regions for discriminating elements in these two families are located in the coding regions, especially the Gag coding region [28,56]. Here we sampled more than 400 segments from coding-elements belonging to these two families, thus increasing the set of sequences available for investigating the variability of the *Ty1* and *Ty2* families. These analyses focused on the extremities of the coding sequences because they are assumed to be more accurately assembled than the internal sequences. Approximately 300 *Ty* segments were extracted and aligned, corresponding to the first 300 nucleotides downstream and upstream of the LTRs, which have been referred to as TYA300 and TYB300. We performed independent phylogenetic reconstructions using these two sets of sequences (Additional file 9 and Additional file 10). The resulting trees (Figure 4) provide a useful means of displaying not only the sequence diversity but also the distribution of the *Ty* subfamilies in the various strains. For example, they clearly show the lack of full-length *Ty1* observed in RM11 and CLIB215 (Additional file 11: Figure S5).

The TYA300 tree (Figure 4A) reveals how large the *Ty1*’ subfamily is. This divergent subfamily differs from *Ty1* mainly in its variant *TYA* sequence. It was initially described in the strain S288c [28], where it belongs to a minor group consisting of only two potentially active elements. Another 20 copies of potentially active *Ty1*’ members were detected here in the strains FL100, Y9, Y12, YJM320, YJM421, YJM269 and YJM789. In Y12 and YJM269, there are even as many *Ty1*’ elements as canonical *Ty1* elements; whereas no potentially functional *Ty1*’ copies were detected in some of the ‘*Ty* permissive’ strains (SIGMA1278, SK1 and CBS7960). The *Ty1*’ subfamily also includes one of the two *Ty* relics mentioned above. This relic, which lacks the *TYB* gene and the 3′ LTR, was detected on chromosome IV (coord. 800,000) of 15 of the strains investigated, some of them apparently devoid of active *Ty1’* copies. This particular *Ty1*’ copy was therefore produced prior to the separation of these strains. Altogether, these findings support for strain specific extinctions or amplifications of this *Ty* variant to have occurred.

The TYA300 tree shows the presence of two clusters that do not include any elements belonging to the reference strain S288c and therefore correspond to new *Ty1* variants. The variant we have called *Ty101* is related to *Ty1*’ (89% identity). The corresponding element was detected in 17 strains at the same location (around position 999,000 on chromosome IV), whereas a *Ty2*-*Ty1* tandem is present at this position in S288c. This variant is the second of the degenerate non-functional *Ty* elements mentioned above: it has undergone chromosomal rearrangements resulting in the loss of *TYB*, and the *TYA* sequence is preceded by an LTR with the opposite orientation. The pattern of organization and the sequence of this variant are highly conserved among the 17 strains (99.5% identity). It is worth noting that this fossil element harbors a single nucleotide substitution at the primer binding site. It should therefore be possible to initiate reverse transcription by using the acceptor stem of the tRNA encoded by tT(UGU)H rather than the tRNAs encoded by the *IMT* genes. The other variant, which we have called *Ty102*, shows 92% identity with the TYA300 from *Ty1* elements and a set of specific SNPs (Additional file 9). One copy of this variant is present in seven strains (CBS7960, FL100, T73, Y10, YJM269, YJM280 and YJM653) at various genomic locations, which suggests that unlike *Ty101*, it is still transpositionally active.

Like the TYA300 tree, the TYB300 tree (Figure 4B) shows the existence of a clear-cut phylogenetic separation between the *Ty1* and the *Ty2* sequences. However, the two clades do not suffice to be able to differentiate accurately between all the *Ty1* and *Ty2* elements: several annotated *Ty1* elements belonging to the strain S288c are included in two clusters containing *Ty2*. These elements correspond to the *Ty1*/*2* hybrids previously described [56]. There are two types of hybrids: those with a *Ty2* TYB terminal segment which is longer than 300 pb and could not be distinguished here from real *Ty2* elements, and ‘short’ hybrids with a 60-pb long *Ty2* segment. The latter elements belong to a distinct cluster containing seven S288c elements and 14 elements belonging to other strains: eight SIGMA1278 elements, three YJM789 elements and one FL100, one CLIB324 and one YJM320 element. It is worth noting that a few additional strains, some of which are apparently not closely related to S288c, were found to carry hybrid *Ty1/2* elements, which indicates that *Ty2* sequences hitchhiking *Ty1* elements and thus enabling them to propagate is not just an oddity which is specific to S288c. Further investigations are now required to check whether hybrid elements of this kind are specific to a whole subset of strains.

The present findings confirm the complexity of the structure of the *Ty1-* and *Ty2*-related subfamilies. They show the existence of considerable diversity, in the form of hybrid elements and divergent subfamilies, two of which correspond to newly described variants. *Ty* variants and hybrids may constitute innovations that improve the maintenance of these elements with time and during the evolution of the host. This is consistent with the fact that the *Ty1’* Gag gene is known to have evolved in response to functional constraints [28]. It has also been established that during the ‘life cycle’ of a TE family [6], mutations lead to the occurrence of variant TE copies. Inter-element recombination processes occurring during retrotransposition events have also been found to drive the divergence among related LTR-retrotransposons [54,57]. This raises questions about the cohabitation in the same genome, of these variants sharing components of both TE and cellular origin. In this context, it is rather striking that *Ty101* is not a currently successful element because reverse-transcription priming of this element by a distinct tRNA might have been expected to provide it with a selective advantage toward *Ty1, Ty2* and *Ty3*. In addition, the patchy distribution of *Ty1’* and *Ty102* may reflect the fact that they encounter variable levels of success, possibly depending on the background of the strain. As regards the origins of the various *Ty1* variants, it is of particular interest that based on the *TYA* sequence, *Ty1* and *Ty1’* were found to be as divergent as *Ty1* and *Ty2* elements; whereas the coding elements belonging to the *Ty3*, *Ty4* and *Ty5* families show much less diversity (data not shown). As in the case of the *Ty2* family, this finding suggests that a horizontal transfer process may have been responsible for the origin of *Ty1’* rather than a process of speciation taking place within the same host.

## Conclusions

Based on the whole genome data available for various *S. cerevisiae* strains, an initial overall picture of the intra-specific genetic diversity of this important model organism was compiled in this study, focusing on its *Ty* retrotransposon content. The results presented here show the considerable differences, which exist between these strains in terms of the number of full-length *Ty* elements, which may in turn act on the future variability of the strains. Some of the strains investigated were found to show considerable insertion polymorphism. As *Ty* insertions are known to alter the rates of expression of adjacent genes [58-61], it would be worth performing further studies in order to assess the potential impact of this polymorphism on the phenotypic characteristics of these strains. Finally, the differences observed here in the composition of the *Ty*1 subfamilies may be attributable to differences between the strain dependent *Ty* maintenance strategies involved. This initial approach was necessary to be able to further investigate and understand the effects of *Ty* elements on the *S. cerevisiae* genome and the interactions between these elements, which govern the equilibrium between *Ty* loss and expansion.

One of the main problems which still remain to be solved is that of the assembling of the large repetitive sequences of which TEs consist. It was not possible here to determine the differences between strains in terms of the mutated and potentially non-autonomous full-length *Ty* elements they contain. However, several studies have shown that processes of competition and complementation between autonomous and non-autonomous TE elements may play an important role in TE dynamics [6,54,57,62]. Recent and still ongoing progress in high throughput sequencing methods may soon make it possible to perform routine sequencing on long reads with a view to assembling these long repetitive sequences without any need for laborious manual finishing. Another important topic that we will then be able to address is the resolution of *Ty*-related gross chromosomal rearrangements such as translocations in the genomes of each strain and their contribution to the diversity and evolution of *S. cerevisiae*.

## Methods

### Strains and genome assemblies

The geographical and ecological origins and references relating to the genome assemblies of the 41 strains investigated here are presented in Table 1. Further information about the surveyed genome assemblies are summarized in Additional file 5: Table S2.

### *Ty* coding-element detection

Sequences containing *Ty* were detected in the genomic assemblies of 41 strains by performing similarity searches with the BLAST suite of programs [63]. *Ty* segments corresponding to the five *Ty* families were identified independently using query sequences from typical full-length elements (Additional file 1).

This first round of searches did not make it possible to discriminate between elements from the *Ty1* and *Ty2* families. In addition, in the 288c reference sequences, 18 out of the 32 full-length *Ty1* elements are in fact *Ty1*/*2* hybrids presumably resulting from recombination events occurring during reverse transcription processes in heterozygous virus-like particles [56]. These hybrid elements have inherited their TyB segment and their 240 bp long LTR-U3 segments from *Ty2*. The sequences detected with the *Ty1* and *Ty2* queries were therefore compared with a set of sequences from thoroughly characterized *Ty1* and *Ty2* elements, excluding the hybrid elements (Additional file 1). The best alignment score was used to assign the sequence affiliation to the *Ty1* or *Ty2* family. Importantly, in the cases where only the LTR or the 3′ extremity of an element were detected, the fact that *Ty2* sequences can be propagated by both *Ty1-* and the *Ty2*-mediated processes makes it impossible to distinguish between *Ty2* elements and *Ty1*/*2* hybrids.

### Mapping *Ty* coding-elements

The regions (2,500 nt long) flanking the *Ty* elements detected were retrieved from the assemblies of the strains investigated. The Repeat Masker program (http://www.repeatmasker.org.) was used to mask *Ty*-related sequences in order to map these flanking regions unambiguously along the S288c reference genome by performing similarity searches. The distributions of the *Ty* elements detected in each strain were compared in order to detect the existence of loci common to several strains as well as specific/singly occupied loci. These data were used to generate “presence/absence matrices” with which to construct heat maps with the R package. Sequences and coordinates of tRNA and RNA polymerase III transcribed genes were downloaded at http://yeastmine.yeastgenome.org/ (06/2012).

### Sequence analyses

The search results were parsed using dedicated python scripts. Multiple sequence alignments were performed with ClustalW2 [64]. Phylogenetic trees were drawn up using the neighbor-joining method (with the Hasegawa-Kishino-Yano 85 substitution model) with Seaview [65]. The trees were then drawn with FigTree (http://tree.bio.ed.ac.uk/software/figtree/).

### Availability of supporting data

The data sets supporting the results of this article are included within the article and its additional files.

## Competing interests

The authors declare that they do not have any competing interests.

## Authors’ contributions

AF and CBG designed the experiments and analysed the data. AF performed the experiments. CBG and JS wrote the manuscript. JS planned the study, participated in its design and acted as the coordinator. All the authors have read and approved the manuscript.

## Supplementary Material

Additional file 1 Query sequences used in similarity searches. Click here for file

Additional file 2: Table S1 Number of total LTRs and LTRs from *Ty* coding-elements per strain. Click here for file

Additional file 3: Figure S1 Distributions of LTR contents in the 41 strains. Boxplots representing distributions of LTR contents (total LTRs and LTRs from coding-*Tys*) in the 41 strains. Click here for file

Additional file 4: Figure S2 Correlations between the number of LTR copies and the number of LTRs belonging to *Ty* coding-elements in each strain. Each point corresponds to one of the investigated strains. Click here for file

Additional file 5: Table S2 Characteristics of the genomic assemblies. Click here for file

Additional file 6: Figure S3 Chromosomal locations of the *Ty* insertions. An individual map was drawn up for each *Ty1* to *Ty5* family. The horizontal axes correspond to the 16 concatenated chromosomes. Alternate white and yellow boxes mark out the chromosome boundaries. Along the chromosomes, the vertical bars indicate the position of the loci corresponding to *Ty* insertions. Blue bars correspond to the presence of LTR, and red bars correspond to the presence of *Ty* coding-elements. The size of the bars is proportional to the number of strains carrying a *Ty* insertion at the same locus. Grey bars indicate the position of RNA polymerase III transcribed genes. The arrowheads indicate the *Ty1* relic copies. Click here for file

Additional file 7 Presence/absence matrices. Click here for file

Additional file 8: Figure S4 Differences between strains in the locations of *Ty* coding-element insertions. *Ty* coding-element insertion profiles in families *Ty1* to *Ty5*: in each strain, each grey rectangle indicates the presence of a *Ty* insertion at the corresponding locus. Dark grey rectangles indicate insertions in common with the S288c reference strain. Hierarchical clustering was applied to both the strains and the loci. The resulting trees are presented in the case of the strains. Click here for file

Additional file 9 TYA300 multiple alignments. Click here for file

Additional file 10 TYB300 multiple alignments. Click here for file

Additional file 11: Figure S5 Distribution of strain sequences in the TYA300 and TYB300 trees. Distribution of RM11 (purple) and CLIB215 (blue) sequences in TYA300 (A) and in TYB300 (B) trees. The arrowheads indicate the *Ty1’* relic copies. Click here for file
