# Supplementary material for: Genome-wide analysis of intraspecific transposon diversity in yeast
Source: BMC Genomics. 2013 Jun 14;14:399. doi: 10.1186/1471-2164-14-399 (PMC4022208; doi:10.1186/1471-2164-14-399)
Supplement: Additional file 2: Table S1 — Number of total LTRs and LTRs from Ty coding-elements per strain. [file 1471-2164-14-399-S2.doc]

|  | **All LTRs** | | | | | | |  | **LTRs from coding-elements** | | | | | |
| --- | --- | --- | --- | --- | --- | --- | --- | --- | --- | --- | --- | --- | --- | --- |
| **Strain** | **Total** | **Ty1** | **Ty2** | **Ty3** | **Ty4** | **Ty5** | **Pol III^a^** |  | **Total** | **Ty1** | **Ty2** | **Ty3** | **Ty4** | **Ty5** |
| AWRI1631 | **227** | 152 | 23 | 26 | 21 | 5 | 150 |  | **1** | -* | - | - | - | 1 |
| AWRI796 | **311** | 181 | 57 | 48 | 22 | 3 | 261 |  | **5** | 3 | 2 | - | - | - |
| CBS7960 | **340** | 195 | 59 | 50 | 28 | 8 | 247 |  | **29** | 11 | 12 | 3 | 3 | - |
| CLIB215 | **296** | 157 | 66 | 44 | 23 | 6 | 235 |  | **26** | 1 | 23 | - | - | 2 |
| CLIB324 | **297** | 182 | 46 | 38 | 26 | 5 | 197 |  | **18** | 8 | 5 | 3 | 2 | - |
| CLIB382 | **148** | 86 | 24 | 20 | 15 | 3 | 32 |  | **1** | 1 | - | -* | - | - |
| EC1118 | **334** | 182 | 74 | 47 | 24 | 7 | 278 |  | **16** | 3 | 10 | - | 2 | 1 |
| FL100 | **366** | 194 | 80 | 46 | 37 | 9 | 225 |  | **33** | 9 | 17 | 3 | 2 | 2 |
| FOSTERSB | **315** | 184 | 61 | 41 | 25 | 4 | 267 |  | **3** | 2 | 1 | - | - | - |
| FOSTERSO | **362** | 206 | 79 | 46 | 26 | 5 | 273 |  | **20** | 6 | 14 | - | - | - |
| I14 | **360** | 195 | 56 | 67 | 32 | 10 | 193 |  | **3** | 2 | - | - | - | 1 |
| IL01 | **277** | 173 | 34 | 43 | 21 | 6 | 109 |  | **1** | - | 1 | - | - | - |
| JA291 | **222** | 159 | 23 | 13 | 18 | 9 | 125 |  | **1** | -* | - | -* | -* | 1 |
| LALVINQA23 | **333** | 184 | 63 | 54 | 23 | 9 | 244 |  | **12** | 4 | 4 | 1 | - | 3 |
| M22 | **191** | 132 | 21 | 12 | 22 | 4 | 83 |  | **2** | -* | - | -* | - | 2 |
| NC02 | **154** | 102 | 21 | 11 | 13 | 7 | 43 |  | **-** | -* | - | -* | - | - |
| PW5 | **167** | 116 | 17 | 10 | 18 | 6 | 94 |  | **-** | -* | - | - | - | - |
| RM11 | **314** | 175 | 67 | 45 | 24 | 3 | 263 |  | **24** | 1 | 22 | - | - | 1 |
| S288C | **406** | 230 | 89 | 41 | 38 | 8 | 279 |  | **99** | 45 | 42 | 4 | 6 | 2 |
| SIGMA1278 | **369** | 221 | 75 | 40 | 26 | 7 | 258 |  | **86** | 45 | 36 | 2 | - | 3 |
| SK1 | **463** | 256 | 111 | 55 | 32 | 9 | 311 |  | **14** | 9 | 4 | - | - | 1 |
| T73 | **147** | 88 | 17 | 20 | 17 | 5 | 89 |  | **3** | -* | - | - | 1 | 2 |
| T7 | **304** | 188 | 44 | 25 | 39 | 8 | 222 |  | **5** | 3 | - | - | - | 2 |
| UC5 | **188** | 143 | 13 | 11 | 18 | 3 | 116 |  | **-** | -* | - | -* | -* | - |
| VIN13 | **328** | 180 | 63 | 53 | 22 | 10 | 259 |  | **9** | 3 | 2 | - | - | 4 |
| VL3 | **306** | 177 | 54 | 50 | 24 | 1 | 268 |  | **2** | - | - | -* | 2 | - |
| WE372 | **350** | 194 | 62 | 68 | 22 | 4 | 136 |  | **3** | 1 | - | - | - | 2 |
| Y10 | **244** | 145 | 45 | 25 | 26 | 3 | 137 |  | **21** | 11 | 6 | 1 | 3 | - |
| Y12 | **380** | 243 | 49 | 35 | 46 | 7 | 244 |  | **27** | 17 | - | 2 | 7 | 1 |
| Y9 | **291** | 180 | 36 | 30 | 40 | 5 | 218 |  | **19** | 10 | 2 | 2 | 5 | - |
| YJM269 | **326** | 193 | 55 | 34 | 37 | 7 | 218 |  | **35** | 14 | 12 | 3 | 4 | 2 |
| YJM280 | **457** | 251 | 99 | 59 | 35 | 13 | 177 |  | **14** | 12 | - | - | - | 2 |
| YJM320 | **393** | 223 | 62 | 64 | 37 | 7 | 226 |  | **6** | 2 | 1 | 1 | - | 2 |
| YJM326 | **381** | 211 | 67 | 69 | 31 | 3 | 172 |  | **2** | 1 | - | - | - | 1 |
| YJM421 | **425** | 226 | 88 | 73 | 34 | 4 | 242 |  | **4** | 2 | 1 | - | - | 1 |
| YJM428 | **208** | 128 | 36 | 18 | 21 | 5 | 69 |  | **2** | 2 | - | -* | -* | - |
| YJM451 | **236** | 166 | 24 | 20 | 18 | 8 | 90 |  | **2** | -* | - | -* | -* | 2 |
| YJM653 | **359** | 204 | 61 | 56 | 31 | 7 | 116 |  | **2** | 1 | - | - | - | 1 |
| YJM789 | **402** | 240 | 83 | 41 | 30 | 8 | 285 |  | **43** | 17 | 24 | - | - | 2 |
| YPS1009 | **208** | 148 | 17 | 17 | 20 | 6 | 93 |  | **18** | 17 | - | -* | -* | 1 |
| YPS163 | **320** | 187 | 47 | 39 | 35 | 12 | 188 |  | **9** | 2 | - | 5 | 2 | - |

* TYA and/or TyB sequences detected but not assembled

^a^ Copy number with RNA polymerase III transcribed gene detected in the flanking sequence

**Table S1. Copy number of LTRs and LTRs belonging to coding-elements**
