# Supplementary figures and images for: Genome-wide analysis of intraspecific transposon diversity in yeast
Source: BMC Genomics. 2013 Jun 14;14:399. doi: 10.1186/1471-2164-14-399 (PMC4022208; doi:10.1186/1471-2164-14-399)

**Figure S1.**

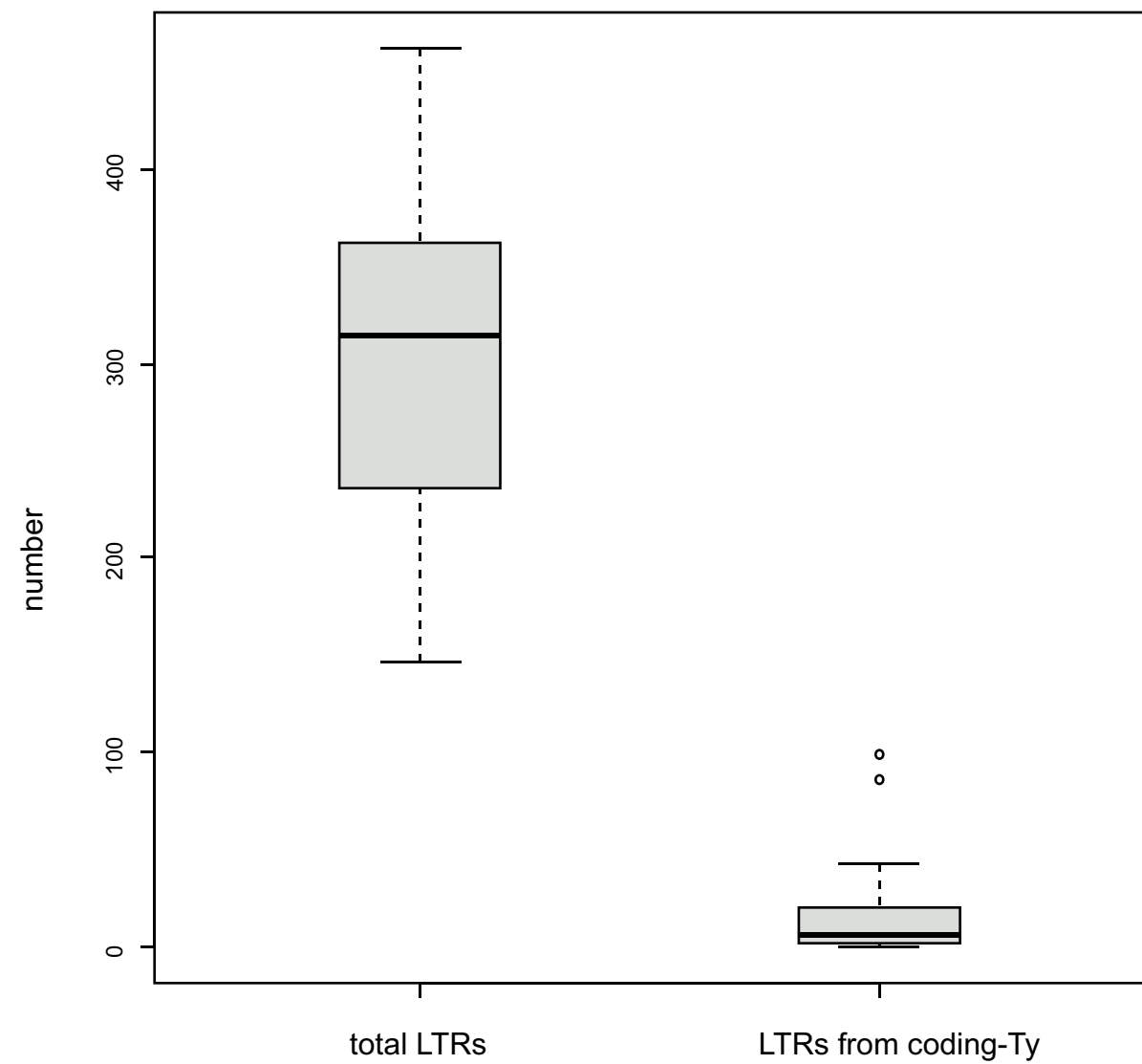

Supplement: Additional file 3: Figure S1 — Distributions of LTR contents in the 41 strains. Boxplots representing distributions of LTR contents (total LTRs and LTRs from coding-Tys) in the 41 strains. [file 1471-2164-14-399-S3.pdf]

**Figure S2.**

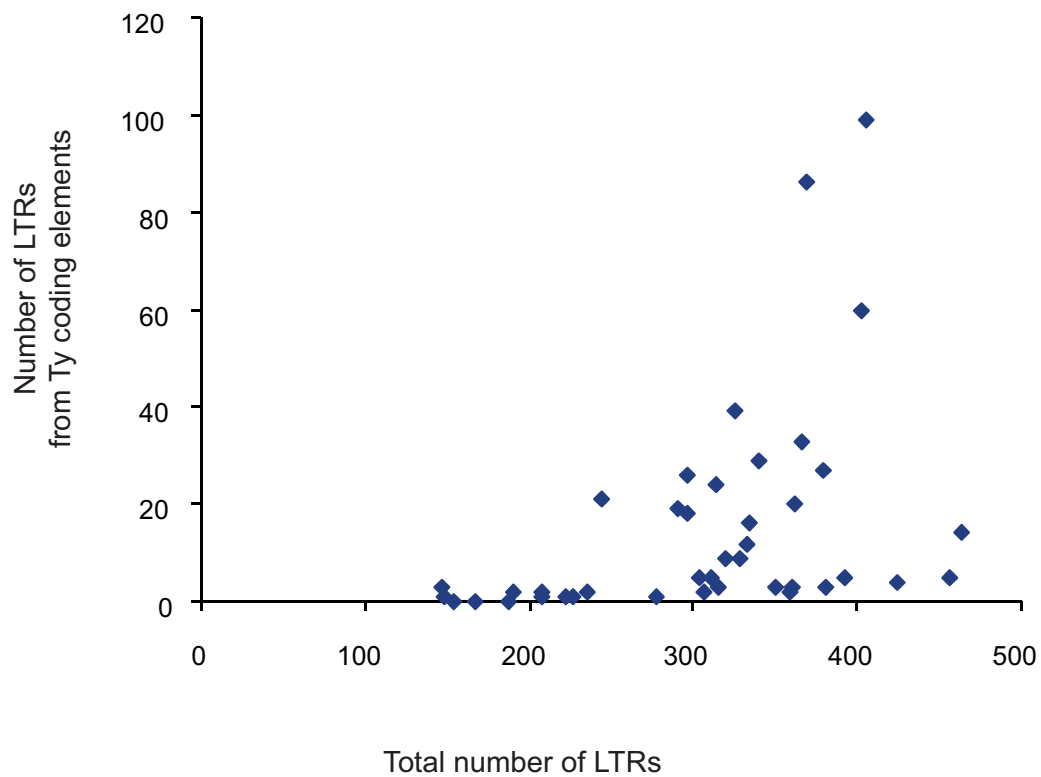

Supplement: Additional file 4: Figure S2 — Correlations between the number of LTR copies and the number of LTRs belonging to Ty coding-elements in each strain. Each point corresponds to one of the investigated strains. [file 1471-2164-14-399-S4.pdf]

Figure S3.

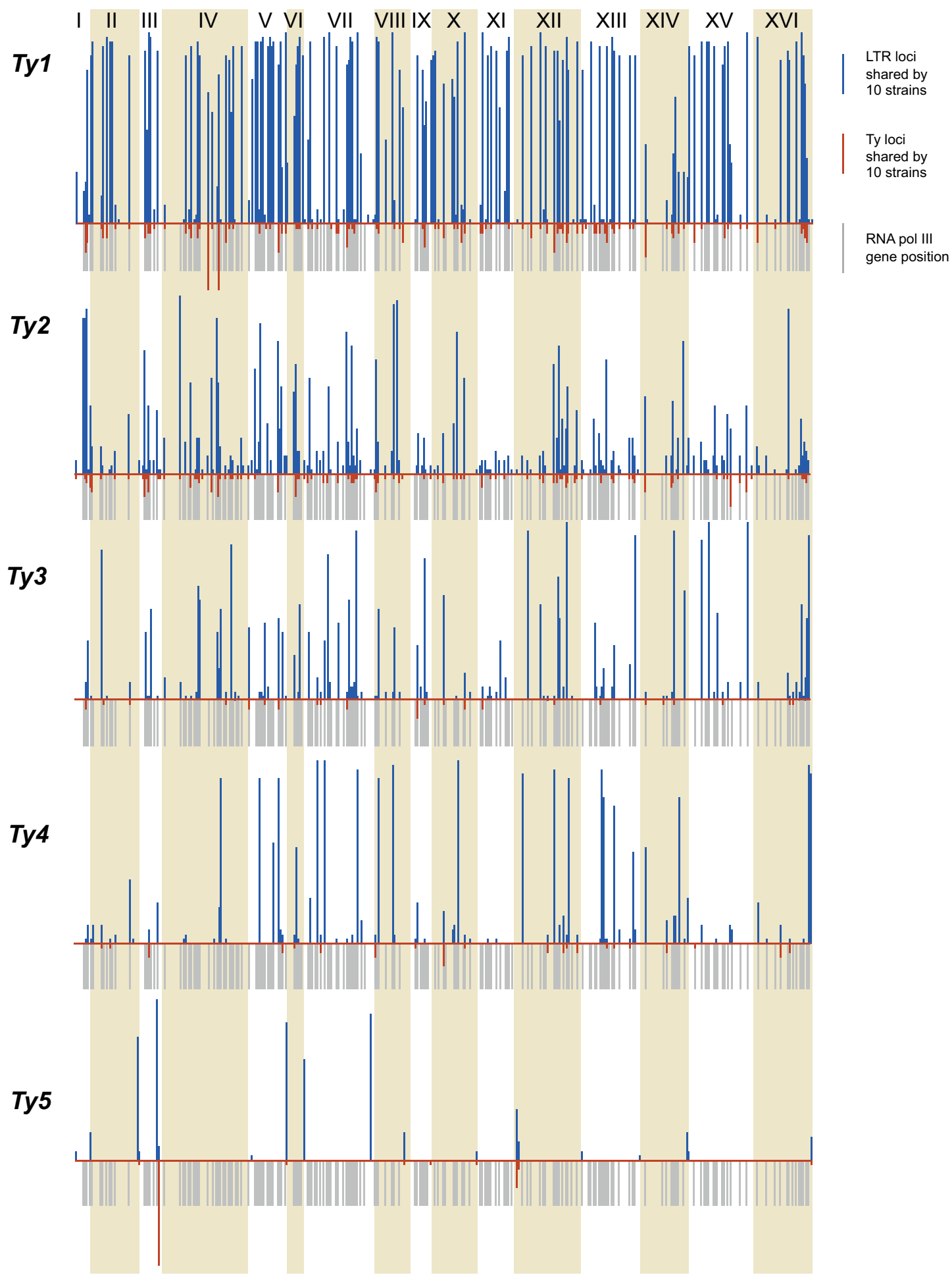

Supplement: Additional file 6: Figure S3 — Chromosomal locations of the Ty insertions. An individual map was drawn up for each Ty1 to Ty5 family. The horizontal axes correspond to the 16 concatenated chromosomes. Alternate white and yellow boxes mark out the chromosome boundaries. Along the chromosomes, the vertical bars indicate the position of the loci corresponding to Ty insertions. Blue bars correspond to the presence of LTR, and red bars correspond to the presence of Ty coding-elements. The size of the bars is proportional to the number of strains carrying a Ty insertion at the same locus. Grey bars indicate the position of RNA polymerase III transcribed genes. The arrowheads indicate the Ty1 relic copies. [file 1471-2164-14-399-S6.pdf]

Figure S4.

Ty1

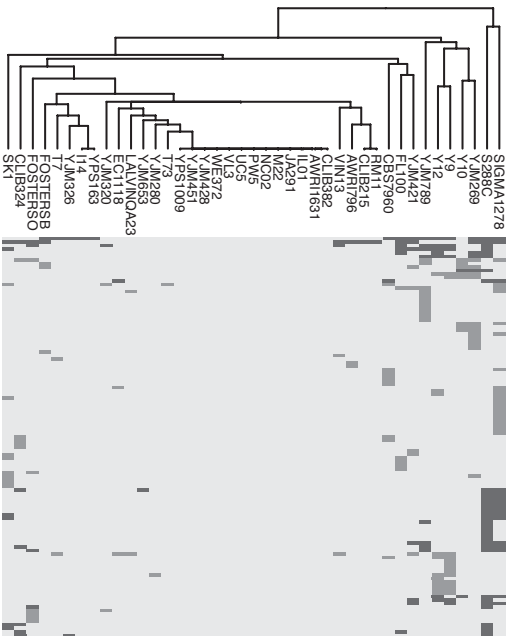

Ty2

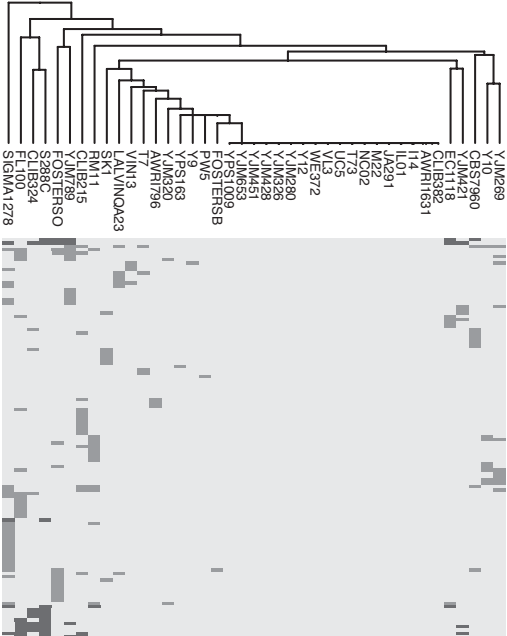

Ty3

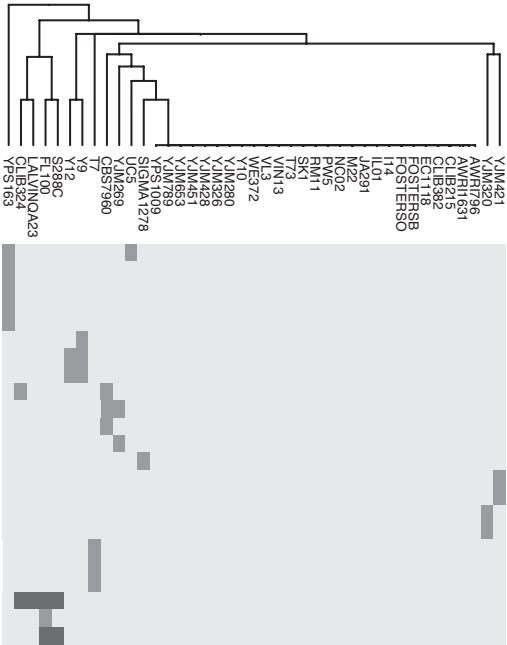

Ty4

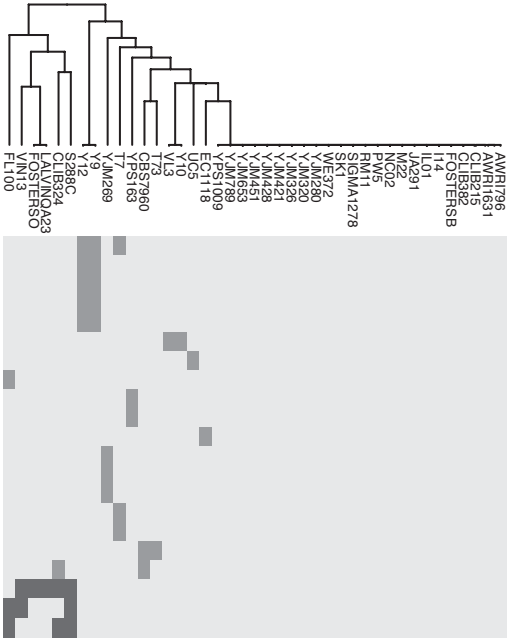

Ty5

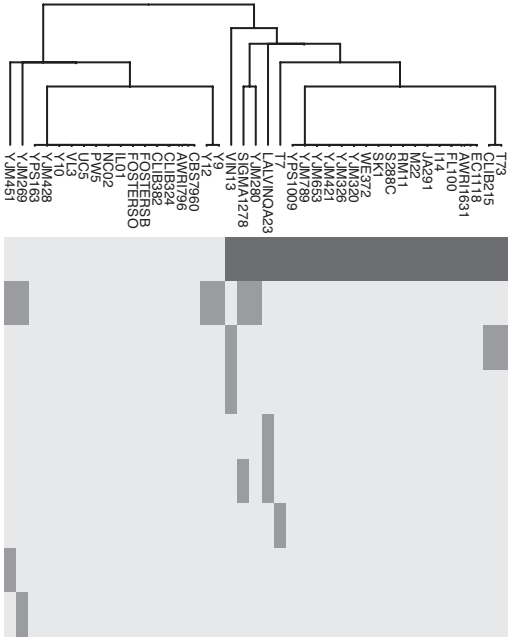

Supplement: Additional file 8: Figure S4 — Differences between strains in the locations of Ty coding-element insertions. Ty coding-element insertion profiles in families Ty1 to Ty5: in each strain, each grey rectangle indicates the presence of a Ty insertion at the corresponding locus. Dark grey rectangles indicate insertions in common with the S288c reference strain. Hierarchical clustering was applied to both the strains and the loci. The resulting trees are presented in the case of the strains. [file 1471-2164-14-399-S8.pdf]

Figure S5.

(A)

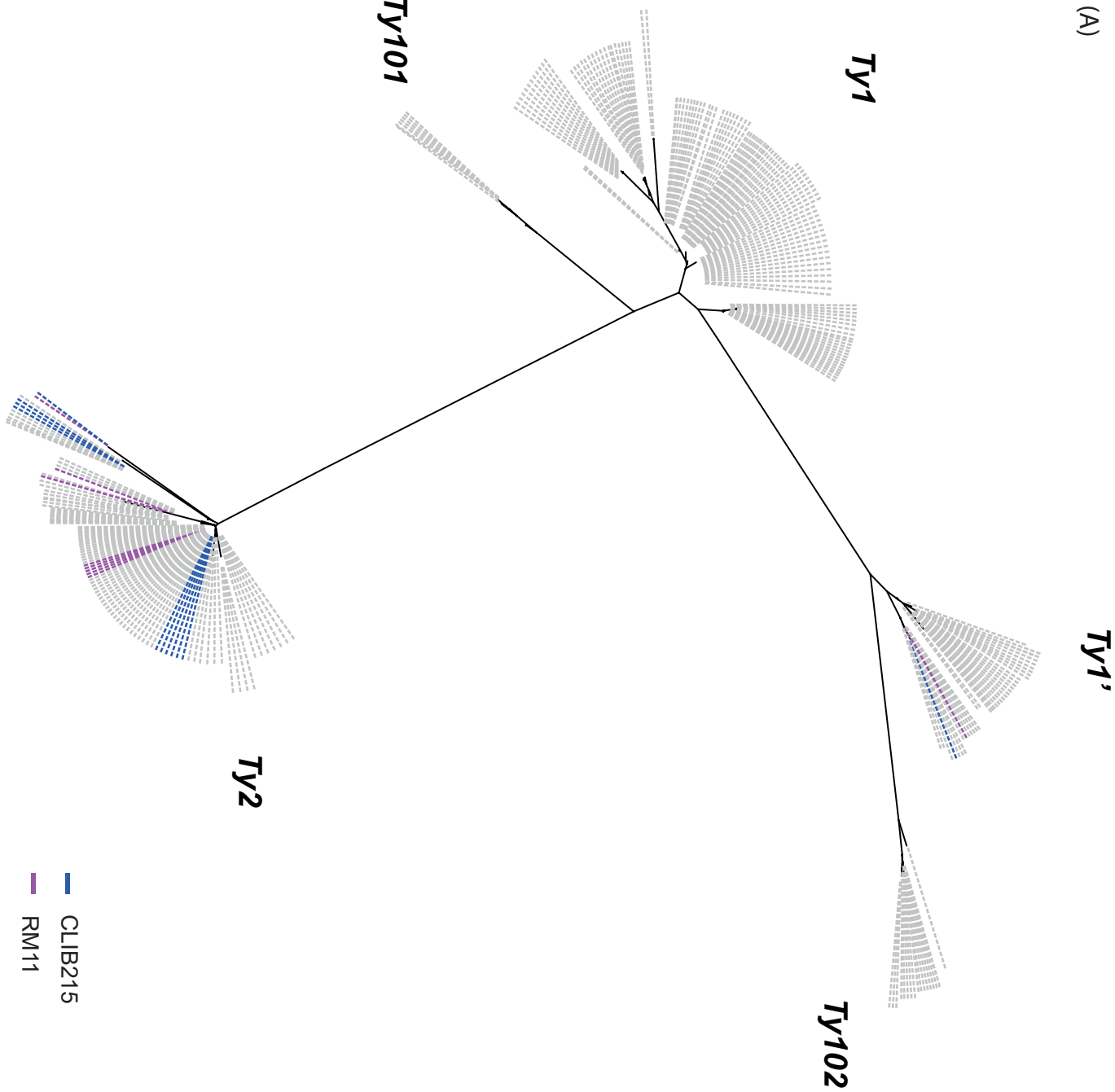

(B)

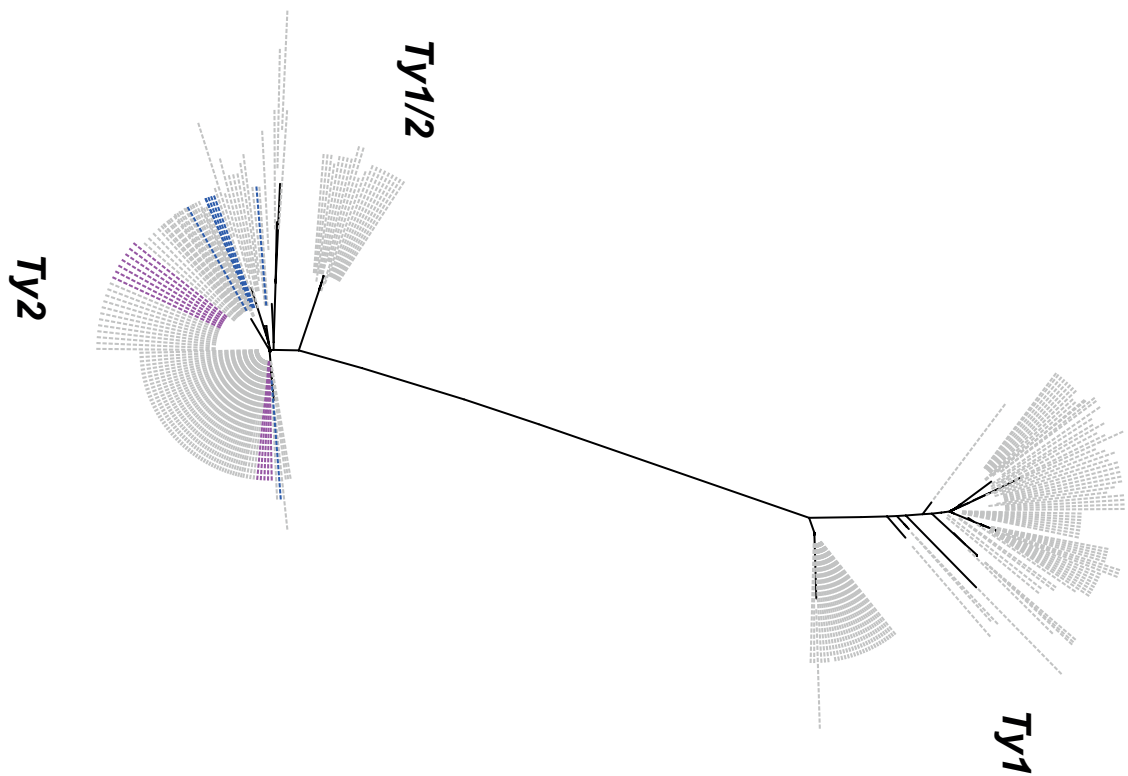

CLIB215  
RM11

Supplement: Additional file 11: Figure S5 — Distribution of strain sequences in the TYA300 and TYB300 trees. Distribution of RM11 (purple) and CLIB215 (blue) sequences in TYA300 (A) and in TYB300 (B) trees. The arrowheads indicate the Ty1’ relic copies. [file 1471-2164-14-399-S11.pdf]
