# Supplementary material for: Genome-wide analysis of intraspecific transposon diversity in yeast
Source: BMC Genomics. 2013 Jun 14;14:399. doi: 10.1186/1471-2164-14-399 (PMC4022208; doi:10.1186/1471-2164-14-399)
Supplement: Additional file 5: Table S2 — Characteristics of the genomic assemblies. [file 1471-2164-14-399-S5.doc]

| Strain | Assembly total size (bp) | Sequencing method | Number of scaffolds | N50 | mean scaffold size (bp) | S288C coverage (%) (BWA) | number of N | LTR | LTR from coding-Ty |
| --- | --- | --- | --- | --- | --- | --- | --- | --- | --- |
| AWRI1631 | 11176855 | 454 | 2484 | 7688 | 4499 | 91,73 | 0 | 227 | 1 |
| AWRI796 | 11459450 | 454 | 16 | 786678 | 716215 | 93,55 | 3222 | 311 | 5 |
| CBS7960 | 11495588 | Sanger* | 244 | 69945 | 47113 | 86,67 | 692506 | 340 | 29 |
| CLIB215 | 11590523 | Sanger* | 340 | 49573 | 34089 | 83,67 | 1056472 | 296 | 26 |
| CLIB324 | 12917159 | Sanger* | 656 | 25610 | 19690 | 81,01 | 2543790 | 297 | 18 |
| CLIB382 | 8589642 | 454* | 12759 | 834 | 673 | 69,61 | 0 | 148 | 1 |
| EC1118 | 11667257 | Sanger and 454 | 38 | 657733 | 307033 | 94,83 | 0 | 334 | 16 |
| FL100 | 11912844 | Sanger* | 594 | 27203 | 20055 | 84,96 | 1026888 | 366 | 33 |
| FOSTERSB | 11254658 | 454 | 16 | 776688 | 703416 | 92,4 | 6118 | 315 | 3 |
| FOSTERSO | 11684932 | 454 | 222 | 194304 | 52634 | 94,55 | 0 | 362 | 20 |
| I14 | 11980221 | Sanger and 454* | 2152 | 21841 | 5567 | 94,78 | 0 | 360 | 3 |
| IL01 | 10858629 | 454* | 7367 | 2249 | 1473 | 87,41 | 0 | 277 | 1 |
| JAY291 | 11537897 | 454 and Illumina | 453 | 64028 | 25469 | 93,51 | 0 | 222 | 1 |
| LALVINQA23 | 11804088 | 454 | 185 | 177377 | 63805 | 94,92 | 0 | 333 | 12 |
| M22 | 11766885 | Sanger and 454* | 3210 | 12613 | 3665 | 93,38 | 0 | 191 | 2 |
| NC02 | 9799542 | 454* | 12884 | 995 | 760 | 78,98 | 0 | 154 | 0 |
| PW5 | 11570262 | 454* | 47 | 393105 | 246175 | 92,27 | 381314 | 167 | 0 |
| RM11 | 11675031 | Sanger | 115 | 228743 | 101522 | 94,71 | 0 | 314 | 24 |
| S288C | 12071326 | Sanger | 16 | 813184 | 754457 | 100 | 0 | 406 | 99 |
| SIGMA1278 | 11906055 | Sanger and Illumina | 67 | 320875 | 177702 | 96,57 | 0 | 369 | 86 |
| SK1 | 12241844 | Illumina | 16 | 804362 | 765115 | 95,66 | 532889 | 463 | 14 |
| T7 | 11092238 | 454* | 43 | 476142 | 257959 | 90,07 | 22562 | 304 | 5 |
| T73 | 11035079 | 454* | 421 | 41276 | 26211 | 80,44 | 1404789 | 147 | 3 |
| UC5 | 11440936 | 454* | 54 | 356094 | 211869 | 92,06 | 323886 | 188 | 0 |
| VIN13 | 11661170 | 454 | 158 | 288574 | 73804 | 95,05 | 0 | 328 | 9 |
| VL3 | 11244008 | 454 | 16 | 781224 | 702750 | 92,37 | 5004 | 306 | 2 |
| WE372 | 11883305 | 454* | 3457 | 7119 | 3437 | 93,74 | 0 | 350 | 3 |
| Y10 | 13872152 | Sanger* | 808 | 22462 | 17168 | 73,59 | 4675809 | 244 | 21 |
| Y12 | 11854261 | Sanger and 454* | 1063 | 82099 | 11151 | 94,59 | 0 | 380 | 27 |
| Y9 | 11036277 | Sanger* | 2324 | 8028 | 4748 | 88,19 | 352 | 291 | 19 |
| YJM269 | 11178560 | Sanger* | 256 | 62877 | 43666 | 83,61 | 711723 | 326 | 35 |
| YJM280 | 11812374 | 454* | 3370 | 7783 | 3505 | 95,06 | 0 | 457 | 14 |
| YJM320 | 11804082 | 454* | 930 | 49176 | 12692 | 94,92 | 0 | 393 | 6 |
| YJM326 | 11681537 | 454* | 1996 | 15097 | 5852 | 94,53 | 0 | 381 | 2 |
| YJM421 | 11816453 | 454* | 1085 | 41297 | 10890 | 95,25 | 0 | 425 | 4 |
| YJM428 | 9924999 | 454* | 10727 | 1256 | 925 | 80,31 | 0 | 208 | 2 |
| YJM451 | 11666438 | 454* | 1390 | 54075 | 8393 | 94,48 | 0 | 236 | 2 |
| YJM653 | 11888871 | 454* | 3864 | 7991 | 3076 | 94,67 | 0 | 359 | 2 |
| YJM789 | 11990995 | Sanger | 258 | 421789 | 46476 | 96,27 | 0 | 402 | 43 |
| YPS1009 | 11406731 | 454* | 3069 | 7410 | 3716 | 92,93 | 15503 | 208 | 18 |
| YPS163 | 11856373 | Sanger and 454* | 1550 | 18562 | 7649 | 94,75 | 0 | 320 | 9 |

* communicated by Justin Fay

**Table S2. Characteristics of the genomic assemblies**
